# Postpartum Stress and the Occurrence of Breastfeeding-Related Spine Musculoskeletal Disorders Among Lactating Mothers in the Dschang Health District, Cameroon

**DOI:** 10.7759/cureus.80604

**Published:** 2025-03-15

**Authors:** Ruslaine Tatuegan Womsi, Hyacinte Trésor Ghassi, Ange Faustine Talla Kenmogne, Julie Annisel Nganmegni Kendjoua, Franklin Chu Buh, Maurice Douryang

**Affiliations:** 1 Department of Biology and Physiology of Animal Organisms, University of Douala, Douala, CMR; 2 Doctoral School, Evangelical University of Cameroon, Bandjoun, CMR; 3 Department of Public Health and Epidemiology, University of Dschang, Dschang, CMR; 4 Department of Physiotherapy, Higher Institute of Foyaguem, Dschang, CMR; 5 Department of Physiotherapy and Physical Medicine, University of Dschang, Dschang, CMR

**Keywords:** health district of dschang cameroon, lactating mothers, postpartum stress, pregnant and lactating, spine musculoskeletal disorders

## Abstract

Background and objective

While postpartum stress (PPS) is a problem generally neglected in the literature, its consequences are devastating for both mothers and their babies. This study aimed to evaluate the role of PPS in the occurrence of spine musculoskeletal disorders among lactating mothers in the Dschang Health District, Cameroon.

Methods

We conducted a cross-sectional study from August 1 to September 29, 2024, involving 422 lactating mothers. Data on PPS were obtained using the Maternal Postpartum Stress Scale (MPSS), and those on the occurrence of breastfeeding-related spine musculoskeletal disorders (BSMSDs) were gathered using the Nordic Musculoskeletal Questionnaire (NMQ). Odds ratios (OR) were calculated, and logistic regression was performed to determine associations. The significance level was set at p<0.05, and the confidence interval (CI) was set at 95%. Stressed women were defined as those with at least a moderate score on the MPSS scale (MPSS score ≥29).

Results

The prevalence rate of BSMSDs in our cohort was 192 (45.5%) with the low back being the most affected area (n=122, 63.5%). The prevalence rate of stress in women was 241 (57.1%). No association was found between PPS and the occurrence of BSMSDs. However, having a secondary scholar-level education [adjusted odds ratio (aOR): 0.57; 95% CI: 0.38-0.87, p˂0.001) and a breastfeeding (BF) session lasting less than 30 minutes (aOR: 0.53; 95% CI: 0.35-0.82; p˂0.001) correlated with a lower incidence of BSMSDs.

Conclusions

Despite the high prevalence of BSMSDs and PPS in lactating women, there was no association between PPS and the occurrence of BSMSDs. Several studies in developed countries have investigated the involvement of stress in the occurrence of MSDs in postpartum women. However, no study to date has evaluated the impact of PPS on the occurrence of spine MSDs among Cameroonian BF women. Data are scarce concerning PPS and the occurrence of spine MSDs, leading to a gap in knowledge and awareness about implementing measures to prevent both PPS and MSDs.

## Introduction

The postpartum period is a time of significant transition for new mothers, as they adjust to the challenges of parenthood. While this phase is often filled with joy, it also brings about heightened stress and a profound sense of responsibility for the mother [1,2]. Health issues that arise during pregnancy or childbirth can persist into the postpartum period, affecting not only the mother's physical, psychological, and social well-being but also the newborn's health [1-4]. The first year following childbirth is especially critical, as it is associated with numerous new experiences and challenges that require the mother to adapt rapidly [5]. One of the most important aspects of this period is breastfeeding (BF), which is considered one of the most effective ways to ensure the health and survival of the infant. In recent years, there has been a strong push to eliminate barriers to early breastfeeding initiation and to promote exclusive breastfeeding, as recommended by global organizations such as the United Nations Children’s Fund (UNICEF) and the World Health Organization (WHO) [6-8]. However, while breastfeeding is widely recognized for its health benefits, it can also be associated with physical health concerns for the mother. Specifically, breastfeeding has been linked to the development of musculoskeletal disorders (MSDs), particularly those affecting the spine, which can significantly impact the mother's overall physical health and well-being [7,9].

The onset of these musculoskeletal issues can often be attributed to factors such as improper breastfeeding positions and poor ergonomics during nursing. As mothers spend prolonged periods in these positions, the strain placed on the spine and surrounding muscles can lead to discomfort and, over time, to more serious musculoskeletal conditions [10-12]. Addressing these risk factors through education on proper positioning, ergonomic adjustments, and self-care practices is critical in mitigating the physical toll that breastfeeding can place on the mother’s body. These interventions are essential not only for the health of the mother but also for promoting a positive breastfeeding experience. Psychological stress is another important factor contributing to the onset of breastfeeding-related musculoskeletal disorders (MSDs) in new mothers. Several studies conducted in developed countries have explored the relationship between postpartum stress and the emergence of physical health problems in women following childbirth [13,14]. Postpartum stress (PPS) is a significant global issue, and it affects new mothers from various cultural and socioeconomic backgrounds [15]. This condition, which includes symptoms of anxiety, depression, and extreme fatigue, not only impacts the mother's emotional well-being but also has the potential to hinder the health and development of the newborn.

The burden of PPS is often intensified by factors such as insufficient support systems, inadequate healthcare, and societal expectations, all of which contribute to both short-term and long-term mental and physical health complications for the mother [4,16]. These stresses can further exacerbate the physical strains of breastfeeding, creating a vicious cycle of discomfort and health issues. To address the challenges posed by postpartum stress, it is crucial to implement comprehensive strategies that include accessible mental health services, strong social support networks, and public awareness campaigns. Such initiatives can help alleviate the effects of postpartum stress, thereby promoting better overall health for both mothers and their children.

Most studies in Sub-Saharan Africa on the psychological conditions of nursing mothers have focused on postpartum depression [3]. However, depression often follows periods of high, unmanaged stress, serving as a heavy burden that can complicate the transition into parenthood [1,17,18]. There are currently no studies from Cameroon on the role of PPS in the onset of breastfeeding-related spine musculoskeletal disorders (BSMSDs). Investigating PPS and its repercussions on physical health will help inform the implementation of strategies to mitigate stress and reduce the occurrence of spine musculoskeletal disorders during BF. Also, the results of this study would serve as a basis for developing policies not only to prevent PPS but also to improve the health of BF women in low and middle-income countries (LMICs) like Cameroon. The aim of this study was, therefore, to determine the role of PPS in the occurrence of BSMSDs in breastfeeding women in the Dschang Health District, in the West Region of Cameroon.

## Materials and methods

Study design and setting

We conducted a cross-sectional study from August 1 to September 29, 2024, in the Health District of Menoua Division, Cameroon, at the Regional Hospital Annex Dschang and the Catholic Center “Notre Dame de la Santé de Batsing’la.” Those settings were used because they are the main and most visited hospitals of the Menoua Division.

Inclusion and exclusion criteria

For participation, patients were required to meet the following specific inclusion criteria: all breastfeeding women from around Menoua Division visiting the Regional Annex Hospital of Dschang and Catholic Center “Notre Dame de la Santé de Batsing’la” who were willing to participate in the study. Participants who did not complete the interview and women with specific types of back pain were excluded from the study.

Participant recruitment

Participants were recruited from the vaccination units of both hospitals. All participants signed an informed consent form, and the current norms for Research on Human Beings were followed after a detailed presentation of the objective of the study. Moreover, written informed consent was obtained from the parent/guardian of each participant aged under 18 years.

The sample size for this study was calculated using Cochran’s formula: N = P(1-P)(Z)^2^/d^2^

Where N = sample size, Z (correspondent of a coefficient interval) at 95% = 1.96 (from the z table), P = expected proportion in the population, which was 50%, which is a standard prevalence, and d = maximum expression error, which is 5%. In the application, we obtained 384 participants. Assuming a potential 10% non-response rate. Four hundred and twenty-two breastfeeding mothers were enrolled.

Data collection/variables

Data were collected through face-to-face interviews with the mothers fulfilling inclusion criteria. The interviews were conducted by a team of trained physiotherapists using a structured questionnaire with three sections (1, 2, and 3). Section 1 elicited data on the participant's sociodemographic and maternal characteristics, including age, occupation, marital status, BMI, child's weight at the time of data collection, and duration of the breastfeeding session. Section 2 sought information on the incidence of spine MSDs and the affected areas using the modified Nordic Musculoskeletal Questionnaire (NMQ) [19], where only the spine regions were considered. The second part of this section evaluated characteristics of pain, such as pain intensity (which was classified as light, moderate, and severe) and painful times (during and after breastfeeding). Finally, we evaluated the incidence of PPS among participants using the Maternal Postpartum Stress Scale (MPSS), which is a reliable tool for assessing self-reported PPS over the previous month [17].

The MPSS has good validity evidence [20]. It comprises 22 items divided into three subscales: personal needs and fatigue, infant nurturing and body changes, and sexuality. The personal needs and fatigue dimension comprises nine items related to adjustment to wake-ups, the baby’s sleep patterns, fatigue, household chores, the lack of help and time for socializing and oneself, the inability to complain, and feelings of loneliness. The infant nurturing dimension includes seven items related to feeding, the baby’s development and health, recognizing the baby’s needs, and soothing a crying baby. The body changes and sexuality dimension consists of six items related to uncertainty about resuming intercourse, the frequency and enjoyment of sexual intercourse, feelings of unattractiveness, and difficulty returning to pre-pregnancy weight and appearance. The participant rates each item on a 5-point Likert scale, ranging from 0 (not experiencing at all) to 4 (experiencing completely). The higher the score on the MPSS, the higher the levels of PPS [17]. 

Statistical analysis

Data were analyzed using IBM SPSS Statistics software, version 23 (IBM Corp., Armonk, NY). Descriptive statistics were used to summarize and quantitatively describe the results, while odd ratios (OR) were calculated, and logistic regression analyses were performed to determine associations between PPS and the occurrence of spine MSDs. The level of significance was set at p<0.05 and a confidence interval (CI) of 95%. Stressed women were defined as those with at least a moderate score on the MPSS scale. Therefore, the prevalence of postpartum stressed women was defined as those with MPSS scores ≥29. This was adapted from the perceived stress scale values [21], as no study to date has proposed a cut-off value for the MPSS. 

Ethical approval

This study adhered to the principles outlined in the Declaration of Helsinki (2013 version, Fortaleza, Brazil). The study received research clearance from the Ethics and Human Health Research Committee, the West Region of Cameroon (no: 785/31/07/2024/CE/CRERSH-OU/VP).

## Results

General characteristics of participants

This study involved 422 postpartum women, with a mean age of 26.9 ± 4.9 years. The mean weight of breastfed children was 7.2 ± 2.6 kg. The majority of the participants (n=250, 59.2%) had a high level of education. The majority (n=265, 62.8%) were married, and the most represented profession among participants was student (n=126, 29.9%). A significant number of the participants were overweight (n=177, 41.9%). Almost all the mothers (n=417, 98.8%) were breastfeeding a single child, and just one woman (0.2%) was breastfeeding three children. The majority of women (n=262, 62.1%) were breastfeeding their children for more than 30 minutes per daily session, and 327 (77%) women had breastfeeding support (Table 1).

**Table 1:** Characteristics of participants and the prevalence of postpartum stress BMI: body mass index; MPSS: Maternal Postpartum Stress Scale

Variables		Frequency (n)	Percentage (%)
Age group, years			
0-26 years		217	51.4
>26 years		205	48.6
Education			
No scholar		3	0.7
Primary		13	3.1
Secondary		156	37.0
Higher level		250	59.2
Marital status			
Single		156	37
Divorced		1	0.2
Married		265	62.8
Profession			
Student		126	29.9
Housewife		85	20.1
Secretary		1	0.2
Other		210	49.8
BMI			
Obese	81		19.2
Normal	164		38.9
Overweight	177		41.9
Duration of daily breastfeeding session, minutes			
15-30	146		34.6
More than 30	262		62.1
Less than 15	14		3.3
Breastfeeding support			
Yes	325		77
No	97		23
Number of children breastfed			
1	417		98.8
2	4		1.0
3	1		0.2
Presence of postpartum stress			
Yes (MPSS score ≥29)	241		57.1
No (MPSS score ˂29)	181		42.9
Total		422	100

Prevalence of postpartum stress

As presented in Table 1, 241 women among the 422 participants had an MPSS score ≥29, showing a postpartum stress prevalence of 241 (57.1%).

Prevalence of breastfeeding-related spine musculoskeletal disorders and pain characteristics

Among the 422 participants, 192 (45.5%) reported postpartum spine musculoskeletal pain. The area most affected was the lower back (n=122, 63.5%), and almost a majority of participants felt pain during and after breastfeeding (n=91, 47.4%). Only 36 (18.7%) women felt severe pain compared to 109 (56.8%) who felt moderate pain (Table 2).

**Table 2:** Spine musculoskeletal pain characteristics among the participants

Presence of spine musculoskeletal disorders	Frequency (n)	Percentage (%)
Yes	192	45.5
No	230	54.5
Affected body part		
Low back	122	63.5
Neck	34	17.7
Neck and low back	36	18.7
Pain intensity		
Severe	36	18.7
Light	47	24.5
Moderate	109	56.8
Time of pain		
After breastfeeding	33	17.2
During and after breastfeeding	91	47.4
During breastfeeding	68	35.4
Total	422	100

Factors associated with the occurrence of spine musculoskeletal disorders

The bivariate analysis revealed no association between PPS and the occurrence of spine MSDs (OR: 0.90; 95% CI: 0.61 -1.32; p=0.60). However, having at most secondary scholar-level education (aOR: 0.57; 95% CI: 0.38 -0.87, p=0.00), a breastfeeding session lasting less than 30 minutes (aOR: 0.53; 95% CI: 0.35-0.82; p=0.00) were found to be protective factors against the occurrence of spine MSDs (Tables 3, 4).

**Table 3:** Association of postpartum stress and sociodemographic variables with spine musculoskeletal disorders *Significant p-value CI: confidence interval; MSD (+): presence of musculoskeletal disorders; MSD (-): absence of musculoskeletal disorders; OR: odds ratio

Variables	Total, n (%) (n=422)	MSD (+), n (%) (n=192)	MSD (-), n (%) (n=230)	OR (95% CI)	P-value
Age group, years					
0-26	217 (51.42)	95 (49.48)	122 (53.04)	0.87 (0.59-1.27)	0.47
>26	205 (48.58)	97 (50.52)	108 (46.96)	-	-
Marital status					
Single/divorced	157 (37.20)	67 (34.90)	90 (39.13)	0.83 (0.56-1.24)	0.37
Married	265 (62.80)	125 (65.10)	140 (60.87)	-	-
Professional activity					
Unemployed (students, housewives)	211 (50.00)	94 (48.96)	117 (50.87)	0.92 (0.63-1.35)	0.69
Inactivity	211 (50.00)	98 (51.04)	113 (49.13)	-	-
Scholar level					
At most secondary	172 (40.76)	66 (34.38)	106 (46.09)	0.61 (0.41-0.90)	0.01*
Superior	250 (59.24)	126 (65.62)	124 (53.91)	-	-
Breastfeeding session duration, minutes					
Less than 30	160 (37.91)	58 (30.21)	102 (44.35)	0.54 (0.36-0.81)	0.00*
More than 30	262 (62.09)	134 (69.79)	128 (55.65)	-	-
Breastfeeding support					
Yes	97 (22.99)	50 (26.04)	47 (20.43)	1.37 (0.87-2.15)	0.17
No	325 (77.01)	142 (73.96)	183 (79.57)	-	-
Postpartum stress					
Yes	241 (57.11)	107 (55.73)	134 (58.60)	0.90 (0.61-1.32)	0.60
No	181 (42.89)	85 (44.27)	96 (41.74)	-	-

**Table 4:** Logistic regression analysis *Significant p-value aOR: adjusted odds ratio; CI: confidence interval; MSD (+): presence of musculoskeletal disorders; MSD (-): absence of musculoskeletal disorders

Variables	Total, n (%) (n=422)	MSD (+), n (%) (n=192)	MSD (-), n (%) (n=230)	aOR (95% CI)	P-value
Scholar level					
At most secondary	172 (40.76)	66 (34.38)	106 (46.09)	0.57 (0.38-0.87)	0.00*
Superior	250 (59.24)	126 (65.62)	124 (53.91)	-	-
Duration of breastfeeding session, minutes					
Less than 30	160 (37.91)	58 (30.21)	102 (44.35)	0.53 (0.35 -0.82)	0.00*
More than 30	262 (62.09)	134 (69.79)	128 (55.65)	-	-

## Discussion

PPS is a common condition among breastfeeding women. This study aimed to evaluate the role of PPS in the occurrence of spine MSDs. Four hundred and twenty-two breastfeeding mothers were enrolled, with a mean age of 26.9 ± 4.9 years. The majority of women (262, 62.1%) were breastfeeding their children for more than 30 minutes per daily session, and 327 (77%) women had breastfeeding support. The prevalence of PPS occurrence was 57%. Spine musculoskeletal pain occurred in 46% with the low back region being the most affected area. Having at most secondary scholar-level education (aOR: 0.57; 95% CI: 0.38-0.87, p=0.00), a breastfeeding session lasting less than 30 minutes (aOR: 0.53; 95% CI: 0.35-0.82; p=0.00) was found to be protective factors against the occurrence of spine MSDs among breastfeeding mothers. The bivariate analysis revealed no association between PPS and the occurrence of spine MSDs (OR: 0.90; 95% CI: 0.61-1.32; p=0.60).

We found a spine MSD prevalence of 45.5%, with the most affected region being the lower back (63.5%). This aligns with the findings of Almutairi et al. [22] in Saudi Arabia, who also reported lower back a the most affected body region. Also, our findings are in line with the results of Ratajczak et al., where the neck was affected at 48% and the lower back at 66% [23]. The higher prevalence of low back involvement could be explained by several factors, such as the postures adopted and the different biomechanical constraints during breastfeeding and caring for the baby. In addition, the physical condition of the participants [the majority of whom were overweight (only 164 of 422 participants had a normal BMI; the rest were either overweight or obese)] could explain the high prevalence of low back pain.

Overweight has been shown in the literature to be a contributing risk factor in the onset of low back pain [24-26]. Other aspects, such as cultural or environmental factors like breastfeeding practices or childcare responsibilities, might also influence this high prevalence of low back pain among lactating mothers in the Dschang Health District. Therefore, education and raising awareness among postpartum women are very important and deserve special attention to reduce the incidence of MSDs of the spine. Of note, our results are at odds with other studies that reported that the neck was the most affected region [7,10]. These differences could be explained by the variations in study designs, as the mentioned studies focused on addressing breastfeeding-related neck pain.

The prevalence of PPS in our population was 57.1%, which is higher than that found among students (31%) [27] and among medical staff (43%) in China [28]. These differences could be explained by the differences in the study setting and population. However, this high rate of stress among breastfeeding women in the Dschang Health District could be explained by several factors, such as traditional expectations around childcare and household responsibilities, lower levels of perceived social support, and inadequate access to quality healthcare, particularly in a rural area like the Menoua Division. This high rate of stress among lactating mothers should attract the particular attention not only of health personnel but also of decision-makers to better protect breastfeeding women and, consequently, their children. Most studies on psychological problems among postpartum women focus on postpartum depression [1,3,18,29]. However, unmanaged stress during the postpartum period can significantly increase the risk of developing more severe mental health conditions, such as postpartum blues, postpartum depression, and postpartum psychosis.

Postpartum blues, characterized by mood swings, irritability, and emotional sensitivity, affects many new mothers but typically resolves within a few days to weeks. However, if stress and emotional distress remain unaddressed, it can progress to postpartum depression, a more serious condition that can impair a mother’s ability to bond with her baby and engage in daily activities. In rare cases, unmanaged stress may lead to postpartum psychosis, a severe mental health emergency that involves symptoms such as hallucinations, delusions, and extreme mood disturbances. These conditions not only threaten the mother’s well-being but can also pose risks to the infant's health and development, underscoring the importance of early intervention and support during the postpartum period [14,29]. It is therefore very important to focus on postpartum stress.

Regarding the association between PPS and the occurrence of spine MSDs, no significant association has been found in this population. To date, no study in Sub-Saharan Africa has investigated the relationship between PPS and spine MSDs among lactating mothers. However, studies in the literature have demonstrated the impact of psychological stress on the occurrence of MSDs [29]. Therefore, we could assume that other factors, such as physical constraints and breastfeeding posture, would be more likely to induce spine MSDs. On another hand, women in the Dschang Health District may employ different stress-coping strategies that may reduce the impact of stress on musculoskeletal health. Prospective studies involving longer follow-up periods should be carried out to better determine the relationship between these two parameters.

A breastfeeding session lasting less than 30 minutes and having at most secondary scholar levels were found to be protective factors against the occurrence of spine MSDs. A short breastfeeding period allows the mother to change position and prevents her from adopting a prolonged breastfeeding position, which is often associated with increased loading of the spine. This allows her musculoskeletal structures to rest. These could be reasons why breastfeeding for less than 30 minutes is a protective factor against spine MSDs. On the other hand, women with lower education levels may be less likely not to have jobs, where work pressure can contribute to stress post-partum, and more likely to engage in traditional physical activities, such as farming or housework, which could promote physical fitness and reduce the risk of MSDs.

Based on the findings of this research, clinical practice should focus on addressing both postpartum stress and spine MSDs in breastfeeding mothers through a multifaceted approach. Education on proper breastfeeding posture and ergonomics is crucial, including training on techniques such as using supportive pillows, alternating breastfeeding positions, and avoiding prolonged static postures. Routine screening for postpartum stress and spinal health should be integrated into maternal health visits, using validated tools to identify issues early. Referral to physical therapists for postpartum-specific exercises, including core strengthening and stretching, can help prevent or manage spine pain.

Stress management interventions, such as mindfulness practices, relaxation techniques, and peer support groups, should also be promoted to improve overall maternal well-being, even though no direct association was found between stress and spine pain. Additionally, a multidisciplinary approach involving obstetricians, physical therapists, lactation consultants, and mental health professionals is essential to provide comprehensive care. For culturally relevant practices in Cameroon, community-based education programs and affordable ergonomic breastfeeding aids should be emphasized, ensuring accessibility and alignment with local needs. Together, these interventions can help mitigate the burden of spine pain and enhance postpartum health.

This study has a few limitations - primarily its cross-sectional design, which does not allow long-term follow-up of the participants. Also, the self-reported pain and stress measures could introduce potential bias. Investigating other factors like breastfeeding position would have helped analyze factors influencing the occurrence of spine MSDs in the Menoua Division. However, despite these limitations, this study is the first of its kind in the African region to investigate the impact of PPS on the occurrence of spine MSDs among lactating mothers.

## Conclusions

This study aimed to investigate the role of PPS in the occurrence of spine MSDs among lactating mothers in the Dschang Health District, Cameroon. Our findings show that the prevalence of spine MSDs was somewhat high, and the most affected region was the lower back. The prevalence of postpartum stress was high in our cohort. No significant association was found between stress and spine MSDs. However, a breastfeeding session lasting less than 30 minutes and having at most secondary scholar-level education were protective factors against spine MSDs. We believe our findings will help inform strategies to reduce PPS to protect mothers from depression and other conditions associated with high levels of stress.
